# Identification of novel markers for neuroblastoma immunoclustering using machine learning

**DOI:** 10.3389/fimmu.2024.1446273

**Published:** 2024-11-04

**Authors:** Longguo Zhang, Huixin Li, Fangyan Sun, Qiuping Wu, Leigang Jin, Aimin Xu, Jiarui Chen, Ranyao Yang

**Affiliations:** ^1^ State Key Laboratory of Pharmaceutical Biotechnology, The University of Hong Kong, Hong Kong, Hong Kong SAR, China; ^2^ Department of Medicine, The University of Hong Kong, Hong Kong, Hong Kong SAR, China; ^3^ Guangdong Provincial Key Laboratory of Food, Nutrition and Health, and Department of Nutrition, School of Public Health, Sun Yat-sen University, Guangzhou, China; ^4^ Department of Clinical Pharmacy, Jining First People’s Hospital, Shandong First Medical University, Jining, China

**Keywords:** biomarker, tumor microenvironment, immunoclustering, machine learning, neuroblastoma

## Abstract

**Background:**

Due to the unique heterogeneity of neuroblastoma, its treatment and prognosis are closely related to the biological behavior of the tumor. However, the effect of the tumor immune microenvironment on neuroblastoma needs to be investigated, and there is a lack of biomarkers to reflect the condition of the tumor immune microenvironment.

**Methods:**

The GEO Database was used to download transcriptome data (both training dataset and test dataset) on neuroblastoma. Immunity scores were calculated for each sample using ssGSEA, and hierarchical clustering was used to categorize the samples into high and low immunity groups. Subsequently, the differences in clinicopathological characteristics and treatment between the different groups were examined. Three machine learning algorithms (LASSO, SVM-RFE, and Random Forest) were used to screen biomarkers and synthesize their function in neuroblastoma.

**Results:**

In the training set, there were 362 samples in the immunity_L group and 136 samples in the immunity_H group, with differences in age, MYCN status, etc. Additionally, the tumor microenvironment can also affect the therapeutic response of neuroblastoma. Six characteristic genes (BATF, CXCR3, GIMAP5, GPR18, ISG20, and IGHM) were identified by machine learning, and these genes are associated with multiple immune-related pathways and immune cells in neuroblastoma.

**Conclusions:**

BATF, CXCR3, GIMAP5, GPR18, ISG20, and IGHM may serve as biomarkers that reflect the conditions of the immune microenvironment of neuroblastoma and hold promise in guiding neuroblastoma treatment.

## 1 Introduction

Neuroblastoma (NBL) is a malignant pediatric tumor originating from neural crest cells, representing the most common extracranial solid tumor in children and accounting for a significant proportion of pediatric cancer cases (1). NBL typically occurs in the adrenal glands but can also develop in nerve tissue along the spine, chest, abdomen, or pelvis. NBL, with its striking predilection for young children, exemplifies the challenges posed by rare malignancies (2). The incidence rate varies across different populations and geographical regions, with NBL being more prevalent in Caucasians than in individuals of African or Asian descent (3). Certain genetic factors, such as mutations in the ALK gene (4), MYCN amplification (5), and familial predisposition (6), are associated with an increased risk of developing NBL. Other factors, including maternal age, birth weight, and prenatal exposures, may also influence NBL development (3). Conversely, NBL is rare in older age groups and adults (7). Due to the significant heterogeneity of NBL (8), MDT (Multi-disciplinary Team) to HIM (Holistic Integrative Medicine) is required to provide patients with precise treatment plans based on the clinicopathological characteristics of the tumor and maximize the clinical benefits for patients. The principal treatments for NBL include systemic chemotherapy and surgery (8, 9). Depending on the disease stage, additional treatment options such as radiotherapy, stem cell transplantation and immune-targeted therapy may be utilized to provide comprehensive care for patients (8, 9). Staging systems for NBL, including the International NBL Risk Group (INRG) (10) classification system and the International Neuroblastoma Staging System (INSS) (11), were developed to standardize the classification of NBL. However, there is still a significant variation in patient outcomes within the same stage group under the current staging system (12). Therefore, further research is needed to investigate the pathophysiological features and classification of NBL within the framework of the existing staging system, to establish a foundation for more precise clinical management and prevent both over-treatment and under-treatment.

The tumor microenvironment (TME) is a dynamic, constantly changing, and highly complex environment composed of tumor cells and a variety of non-tumor cells, including immune cells, fibroblasts, *etc.*, which play a critical role in tumor proliferation, invasion, and metastasis (13, 14). The tumor immune microenvironment (TIME), which constitutes a significant component of the TME, encompasses not only immune cells (*e.g.*, T cells, macrophages, dendritic cells.), but also non-immune cells (*e.g.*, fibroblasts, endothelial cells.), extracellular matrix components, and a range of molecules involved in the immune response (15). The TIME shapes tumor biological features through both anti-tumorigenic and pro-tumorigenic effects. On the one hand, the immune cells in TIME are activated to recognize and eliminate tumor cells (16). Moreover, immune cells have the capacity to generate cytokines and chemokines, which serve to attract other immune cells to the tumor site and stimulate an immune response against the tumor (16, 17). On the other hand, tumor cells can also manipulate the TIME and eventually undermine effective tumor surveillance (18). Tumors achieve immune evasion through multiple pathways (18–20), including dysfunctional antigen presentation mechanism, recruitment of regulatory immune cells, and induction of T cell exhaustion, etc. In non-small cell lung cancer (21) and colorectal cancer (22), tumor typing based on TIME can enhance the prediction of patient prognosis and provide guidance for clinical therapy. In NBL, the immune checkpoint-based signature constructed using OX40, B7-H3, ICOS, and TIM-3 can differentiate between high- and low-risk NBL patients and has the potential to predict prognosis (23). However, there is a lack of sufficient clinical evidence to reveal the relationship between the TIME of NBL and its clinicopathologic features. Furthermore, accurately diagnosing the level of immune infiltration in NBL remains challenging.

**Figure 1:**
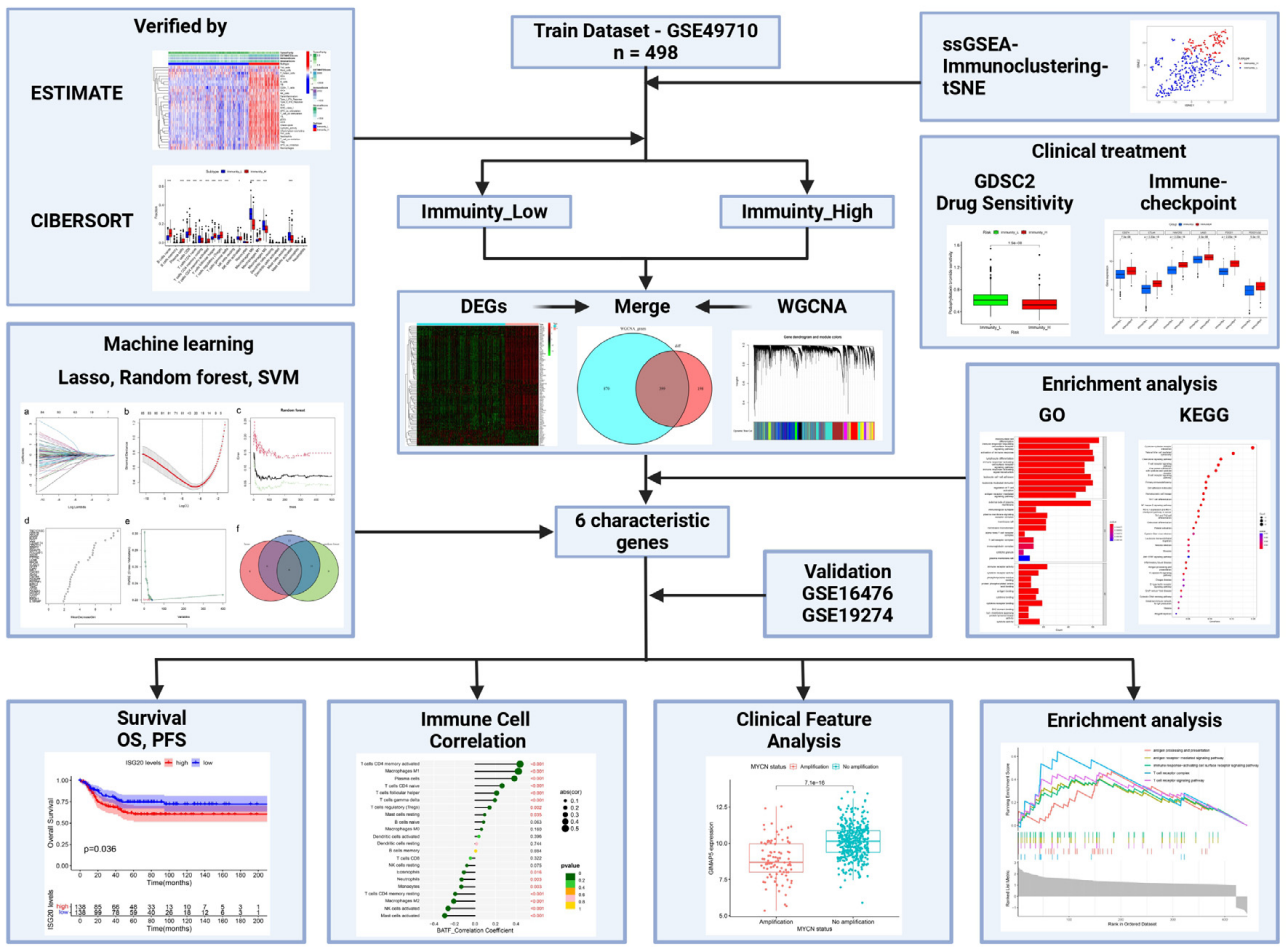
The workflow of this study.

In this study, we calculated the immune enrichment score of each sample using ssGSEA (single-sample Gene Set Enrichment Analysis), and used hierarchical clustering to categorize the samples into high and low immunity groups. Subsequently, we systematically evaluated the relationship between the clinicopathologic features of the tumor and the status of immune infiltration. To better distinguish the immune subset profile of NBL, we employed three machine-learning algorithms to identify potential diagnostic biomarkers and analyze the function of these characterized genes in NBL (**Figure 1**).

## 2 Methods

### 2.1 Data acquisition and processing

All transcriptome sequencing data for NBL were obtained from the Gene Expression Omnibus (GEO) database (https://www.ncbi.nlm.nih.gov/geo/). The GSE49710 dataset [GPL16876 platform, Agilent-020382 Human Custom Microarray 44k (Feature Number version)] containing 498 tumor samples was downloaded for the training set. Two independent cohorts GSE16476 (GPL570 platform, [HG-U133_Plus_2] Affymetrix Human Genome U133 Plus 2.0 Array, n=88) and GSE19274 (GPL6102, Illumina human-6 v2.0 expression beadchip, n=100) were used as test datasets. The GSE85047 dataset (GPL5175 platform, [HuEx-1_0-st] Affymetrix Human Exon 1.0 ST Array [transcript (gene) version], n=283) was used for survival analysis of characterized genes. The probe names in the original gene expression matrix were converted to gene symbols using Perl (v5.32.1.1), and duplicate gene symbols were averaged. The expression information of probes without gene symbols was deleted.

### 2.2 Immunoclustering performed by ssGSEA

As a rank-based algorithm, ssGSEA (single-sample Gene Set Enrichment Analysis) can be conducted to investigate the absolute enrichment levels of each sample in a particular set of genes by calculating its enrichment score (24). In this study, ssGSEA was employed to investigate the enrichment levels of 29 clusters of immune cell markers, or gene sets associated with immune function and/or pathways to reflect the immune infiltration conditions of tumors, in each NBL tissue using “GSVA” (25) and “GSEABase” R packages. Hierarchical clustering analysis was applied to classify NBL tissues into high or low immunity groups based on ssGSEA enrichment results among immune signature genes, using “sparcl” (26) R package.

### 2.3 Validation of immunoclustering

As a dimensionality reduction technique, t-SNE (t-distribution Stochastic Neighbor Embedding) is commonly used to visualize high-dimensional data into low-dimensional data. It excels at capturing complex patterns and relationships within the dataset (27). The t-SNE algorithm was employed, with the assistance of the “Rtsne” R package, to analyze the distribution of NBL samples across different immunity conditions.

Since non-tumor cells and tumor cells display distinct gene expression patterns, the ESTIMATE (Estimation of STromal and Immune cells in MAlignant Tumor tissues using Expression data) algorithm can calculate the relative abundance of non-tumor cells (e.g., immune cells and stromal cells) in tumor tissues by analyzing the expression of characteristic genes among those cells within the sample (28). To assess the outcomes of the ssGSEA-based hierarchical clustering, ESTIMATE was employed to analyze four tumor microenvironment scores including tumor purity, ESTIMATE score, immune score, and stromal score. This analysis was performed for each patient to compare the high and low immunity groups, using “estimate” (29) R package.

The CIBERSORT (Cell-type Identification By Estimating Relative Subsets Of RNA Transcripts) algorithm quantifies the relative proportions of immune cell clusters in tumor samples by deconvolution of bulk RNA-Seq results using a support vector regression (SVR) machine learning approach (30). It assesses the fraction of 22 tumor-infiltrating immune cell (TIIC) subpopulations, including T cells, B cells, natural killer cells, macrophages, etc., in the tumor microenvironment. This assessment was based on the matrix file named “LM22,” which comprises 547 genes and covers 22 mature human hematopoietic populations (31). The abundance of different immune cells between the high and low immunity groups of NBL was investigated using CIBERSORT algorithm.

### 2.4 Therapeutic response analyses

The influence of the immune infiltration level of NBL on clinical drug therapy was analyzed. Firstly, the effect of immunity on NBL immunotherapy was investigated, and we examined six immune-checkpoint-associated genes (CD274, CTLA4, HAVCR2, LAG3, PDCD1, PDCD1LG2) in the high and low immunity groups. Utilizing data from the Genomics of Drug Sensitivity in Cancer 2 database (GDSC2) at https://www.cancerrxgene.org/, the “oncoPredict” (32) R package was used to predict the sensitivity of each patient to different drugs, further use of the Wilcoxon rank-sum test to analyze the differences between high-sensitivity drugs in various immunity conditions.

### 2.5 Differentially expressed gene analysis

Using the “limma” (33) R package to analyze differentially expressed genes (DEGs) between the low and high immunity groups of NBL samples in the log2-processed training dataset. In this process, genes with log2|fold change (FC)| ≥ 1 and adjusted p-value < 0.05 were identified as DEGs affected by immune infiltration levels and were used as candidate genes for further analysis. Meanwhile, for the NBL immune-related characteristic genes identified by machine learning, the samples were categorized into high- and low-expression groups based on the median expression of these genes. The selection method of DEGs between high and low expression groups was the same as described previously.

### 2.6 Weighted gene co-expression network analysis

Weighted Gene Co-Expression Network Analysis (WGCNA) was used to cluster highly synergistic gene modules in NBL, correlate these modules with immunity classifications, and subsequently identify immune-associated core modules and genes. This analysis was performed using the “WGCNA” R package (34). Initially, the log2-processed gene expression matrix was screened to remove genes with low expression levels. To determine the appropriate soft threshold power value for the median calculation of the correlation coefficient to obtain the scale-free network distribution, the “pickSoftThreshold” function is employed to calculate the soft thresholding power (β) value. Subsequently, the “sft$powerEstimate” function is used to select the optimal β value from a range of 1 to 20, with the requirement that the scale-free topology fit R^2 index exceeds 0.85. Adjacency matrix and TOM matrix were then constructed sequentially on the basis of the gene expression matrix according to the β value. The dynamic tree cut algorithm was used to construct modules and analyze the correlation between modules and immunity.

### 2.7 Functional enrichment analysis

Prior to analysis, the symbol IDs of all genes were converted to Homo sapiens EnterzID using the “org.Hs.eg.db” R package. We explored the functions of all DEGs associated with immunity of NBL using Gene Ontology (GO) analysis and Kyoto Encyclopedia of Genomes (KEGG) analysis with the assistance of the “clusterProfiler” (35) R package, and the screening criteria for the terms were p < 0.05. GO gene set enrichment analysis (GSEA) was applied to conduct enrichment analysis of DEGs among high- and low-expression groups of each immune infiltration-related Genes using “clusterProfiler” R package.

### 2.8 Machine learning

Three Machine Learning algorithms were utilized to identify characteristic genes affecting immunity levels in NBL. LASSO (Least Absolute Shrinkage and Selection Operator) possesses inherent regularization properties, allowing it to perform feature selection by shrinking specific regression coefficients to zero. This unique property makes LASSO well-suited for identifying relevant variables and selecting a subset of significant features in a given dataset (36). Genes were ranked by Random Forest, and the top 30 genes were selected for further analysis. SVM-RFE (Support Vector Machine Recursive Feature Elimination) operates as a feature selection algorithm reliant on Support Vector Machines (SVMs). SVM separates different classes of data points in a high-dimensional feature space by finding the optimal hyperplane (37). LASSO, SVM, and Random Forest were implemented using the “glmnet” (38), “e1071” (39), and “randomForest” (40) R packages, respectively. The intersection of LASSO, SVM, and Random Forest results was used to identify hub genes affecting immunity in NBL. Determine the area under the curve (AUC) using the receiver operating characteristic (ROC) curve to evaluate the characterized genes and assess their discrimination value in the immune infiltration levels, using the “pROC” (41) R package.

### 2.9 Survival analyses

In order to investigate the effect of the immunity-related characteristic gene expression on the prognosis of NBL patients, we analyzed the OS (Overall Survival) and PFS (Profression Free Survival) data in the GSE85047 dataset. Samples were categorized into two high and low expression groups based on the median gene expression, and the Kaplan-Meier curve was used to investigate the survival differences between the two groups, with the assistance of the ‘survival’ and ‘survminer’ R packages.

### 2.10 Statistical analysis

Continuous data were presented using mean ± standard deviation (M ± SD), and categorical and count data were expressed as frequencies (rates). When analyzing differences in clinical features between high and low expression groups of characterized genes using IBM SPSS Statistics 27, the Mann-Whitney U test was used for continuous variables and the chi-square test for categorical variables. A two-sided p-value of less than 0.05 was considered to indicate a statistically significant difference. The remaining analysis was performed using R software (version 4.2.2). The Wilcoxon rank-sum test was used to analyze the difference in continuity variables between the Immunity_L and Immunity_H groups. P < 0.05 was considered statistically significant.

## 3 Results

### 3.1 Immunoclustering of NBL

**Figure 2:**
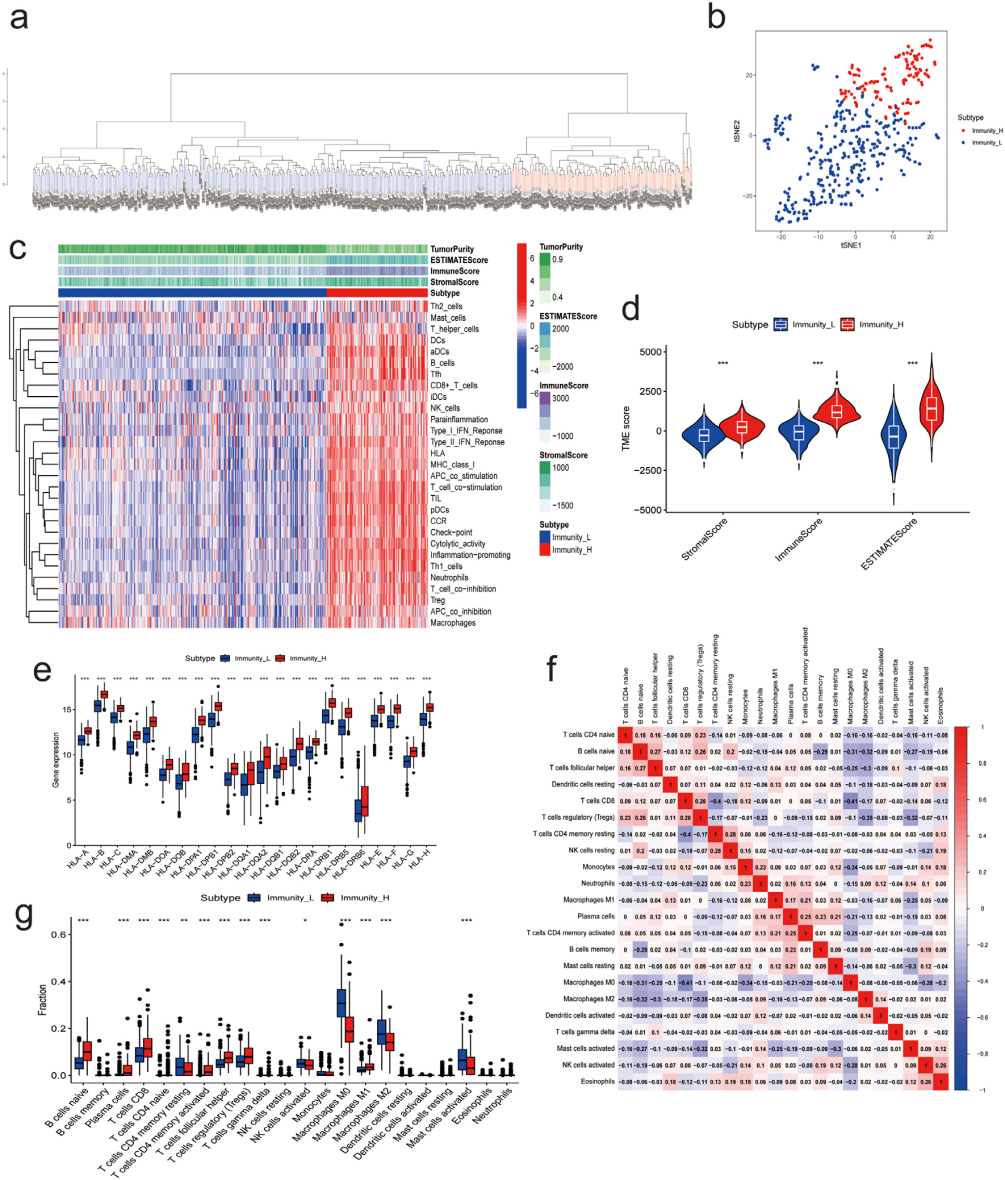
Results of NB Immunoclustering. **(A)** Hierarchical clustering analysis results based on ssGSEA enrichment score. **(B)** t-SNE analysis of the high- and low-immunity groups. **(C)** Heatmap of ESTIMATES analysis results. **(D)** Violin plot of ESTIMATES analysis results. **(E)** Expression of immune-related genes between high and low immunity groups. **(F)** Correlation analysis between 22 immune cells from CIBERSORT analysis. **(G)** Immune cell infiltration between high and low immunity groups. (* P<0.05, ** P<0.01, *** P<0.001).

Scoring the immune infiltration conditions of 498 NBL samples from the training dataset (GSE49710) based on transcriptome sequencing data using the ssGSEA algorithm. An unsupervised hierarchical clustering algorithm identified two clusters with different immune infiltration patterns based on the ssGSEA scores. In total, 362 samples belonged to the low immunity group (Immunity_L), while 136 samples were categorized as the high immunity group (Immunity_H), as illustrated in **Figure 2A**. t-SNE analysis further demonstrated distinct gene expression patterns between the high and low immune infiltration groups (**Figure 2B**). To validate the immunoclustering based on ssGSEA scores, the ESTIMATE algorithm was employed. The results indicated that the high immunity group exhibited significantly higher ESTIMATE scores (L: -448.0756 ± 1095.2302; H: 1452.7437 ± 936.7952), ImmuneScores (L: -146.0297 ± 627.0643; H: 1237.8876 ± 554.5626), and StromalScores (L: -302.0459 ± 559.7807; H: 214.8562 ± 502.2821). Additionally, this group showed lower Tumor Purity (L: 0.8470 ± 0.0784; H: 0.6772 ± 0.1013) (**Figures 2C, D**). Concurrently, multiple HLA genes upregulated in the immunity group, as shown in **Figure 2E**. Furthermore, CIBERSORT results indicated that NBL samples with high immunity exhibited increased levels of B cells naive, Plasma cells, T cells CD8, T cells CD4 naive, T cells CD4 memory activated, T cells follicular helper, T cells regulatory (Tregs), T cells gamma delta, Macrophages M1 infiltration, and lower levels of T cells CD4 memory resting, Macrophages M0, Macrophages M2, and Mast cells activated infiltration (**Figures 2F, G**). The clinicopathologic characteristics among the different groups were shown in **Supplementary Table S1**.

### 3.2 Immunoclustering and clinical treatment

**Figure 3:**
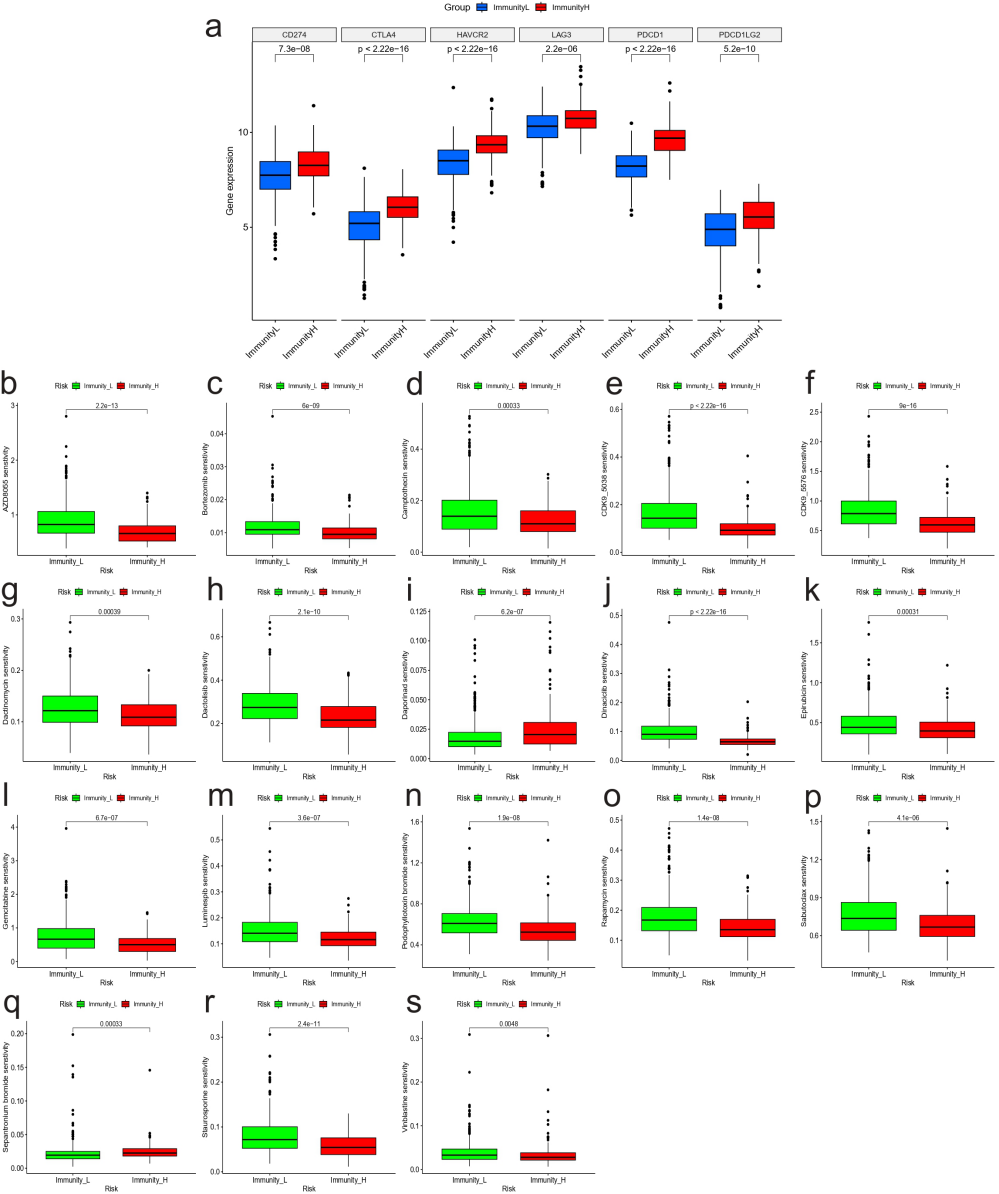
Immunoclustering and clinical treatment results. **(A)** Expression of genes associated with immune checkpoints between high and low immunity groups. Results of drug sensitivity analysis: **(B)** AZD8055; **(C)** Bortezomib; **(D)** Camptothecin; **(E)** CDK9_5038; **(F)** CDK9_5576; **(G)** Dactinomycin; **(H)** Dactolisib; **(I)** Daporinad; **(J)** Dinaciclib; **(K)** Epirubicin; **(L)** Gemcitabine; **(M)** Luminespib; **(N)** Podophyllotoxin bromide; **(O)** Rapamycin; **(P)** Sabutoclax; **(Q)** Sepantronium bromide; **(R)** Staurosporine; **(S)** Vinblastine.

To further investigate the clinical value of different immunity levels in NBL, we analyzed the differences between high and low immunity groups in immunotherapy and chemotherapy. The results showed that the high immunity group had higher expression of 6 immune checkpoint-related genes, as shown in **Figure 3A**. Meanwhile, the drug sensitivity results showed that the high immunity group was more sensitive to drugs AZD8055, Bortezomib, Camptothecin, CDK9_5038, CDK9_5576, Dactinomycin, Dactolisib, Dinaciclib, Epirubicin, Gemcitabine, Luminespib, Podophyllotoxin bromide, Rapamycin, Sabutoclax, Staurosporine, Vinblastine; while the low immunity group was more sensitive to drugs Daporinad, Sepantronium bromide (**Figures 3B–S**).

### 3.3 Identification of immunity-related differentially expressed genes

**Figure 4:**
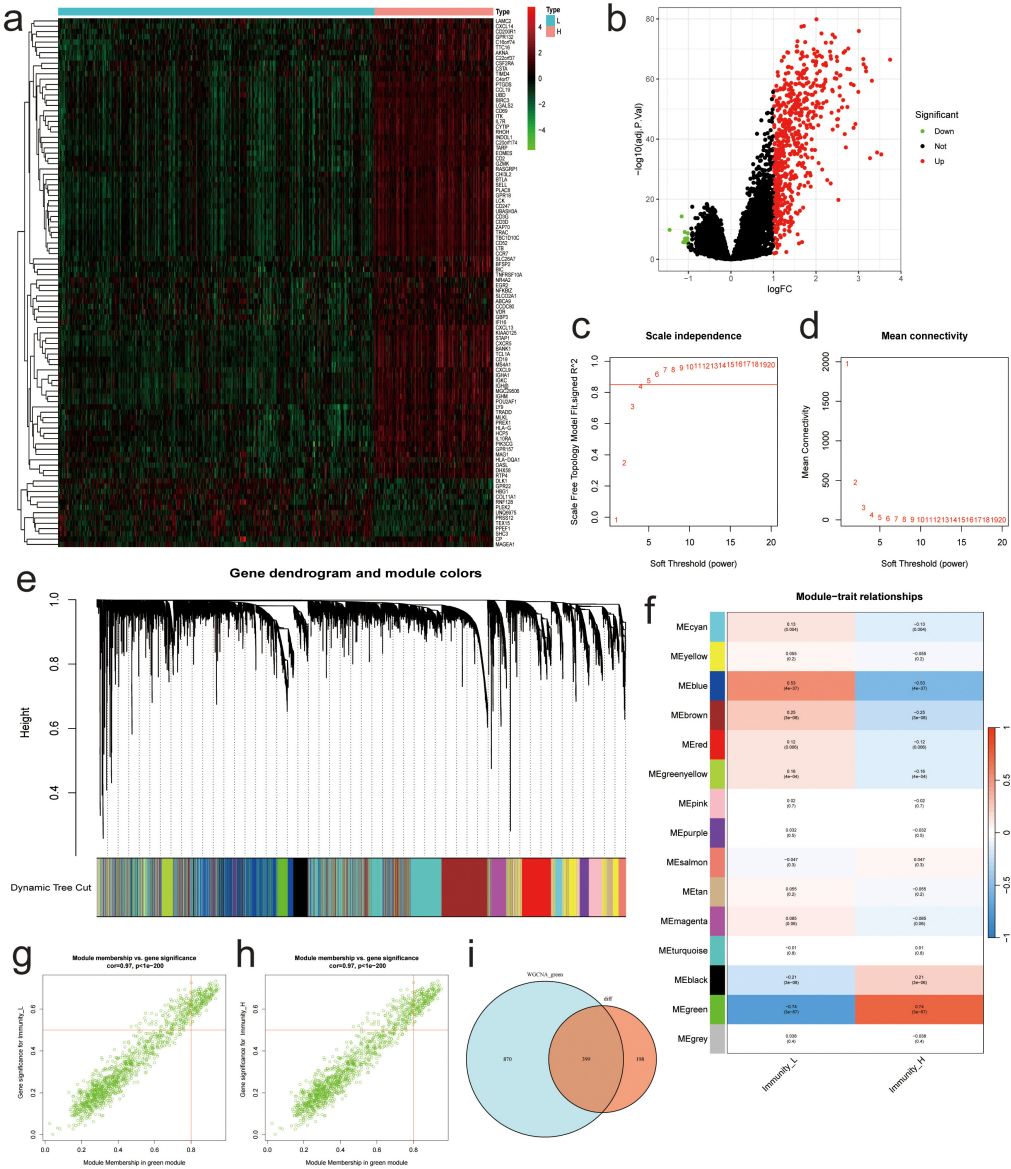
Results of Differential Expression Analysis and WGCNA Analysis. **(A)** Heatmap of the most significantly differentially expressed genes. **(B)** Volcano plot of differentially expressed genes. **(C)** Relationship between β and R^2. **(D)** Plots of β and mean connectedness. **(E)** Clustering tree diagram for all genes. **(F)** Heatmap of the relationship between the characteristic gene modules and immunity. Scatter plots of GS score and MM for each gene in the MEgreen module among **(G)** low and **(H)** high immunity groups. **(I)** Venn diagram showing differentially expressed genes associated with immunity between different groups.

In the training dataset, 586 genes were up-regulated, while 11 genes were down-regulated in the high-immunity NBL group when compared to the low-immunity group based on “limma” method(**Figures 4A, B**). After excluding abnormal samples and genes with low expression, we conducted WGCNA analysis on the remaining samples to identify clusters of genes associated with NBL immunity. The scale-free network was constructed with an R^2 value greater than 0.85, using a soft threshold power of 5 (**Figures 4C, D**). Fifteen genetic modules were identified by dynamic cutting tree, among which the MEgreen module displayed the highest positive association with immunity to NBL (L: R = -0.74, p < 0.001; H: R = 0.74, p < 0.001) (**Figures 4E–H**). After intersecting the sets of differentially expressed genes and MEgreen module genes, a total of 399 differentially expressed genes associated with NBL immunity were identified as candidates for further analysis (**Figure 4I**).

### 3.4 GO and KEGG analysis results

**Figure 5:**
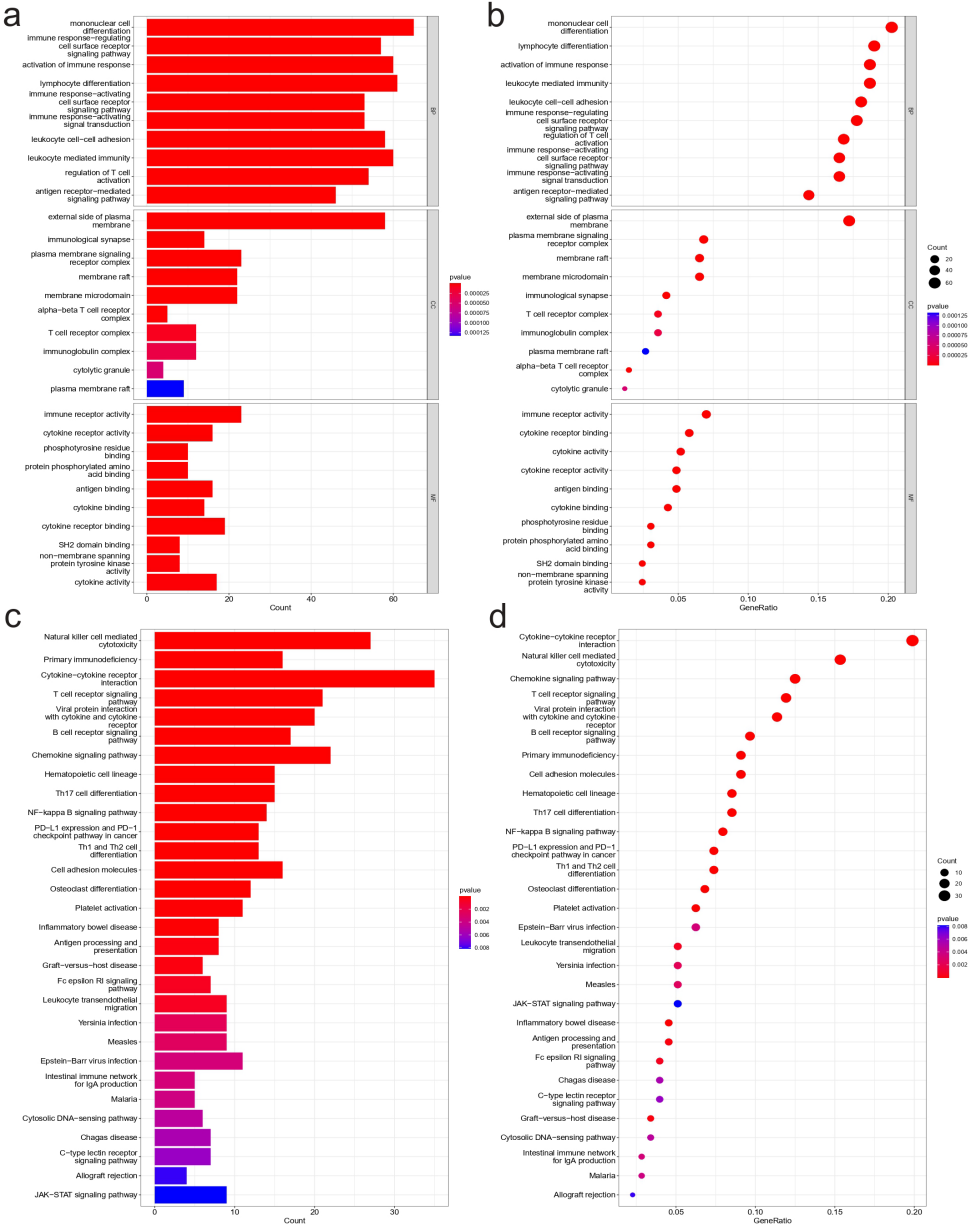
Functional enrichment analysis results. Results of GO analysis: **(A)** Bar plot based on gene count, **(B)** Bubble plot based on gene ratio. Results of KEGG analysis: **(C)** Bar plot based on gene count, **(D)** Bubble plot based on gene ratio.

To further investigate the mechanisms that contribute to the different immune conditions in NBL, enrichment analyses of differentially expressed genes that are positively correlated with immunity in NBL were conducted. The GO results based on gene count (**Figure 5A**) and gene ratio (**Figure 5B**) revealed that the candidate genes were involved in multiple immune-related biological process pathways (*e.g.*, mononuclear cell differentiation, activation of immune response, immune response−regulating cell surface receptor signaling pathway.) and were present in multiple immune-related cellular components (*e.g.*, alpha-beta T cell receptor complex, T cell receptor complex, immunoglobulin complex.), and the molecular functions involved include, for example, immune receptor activity, cytokine receptor activity, antigen binding. KEGG results based on gene count (**Figure 5C**) and gene ratio (**Figure 5D**) also showed that candidate genes were enriched in several immune-related pathways, including Natural killer cell mediated cytotoxicity, Primary immunodeficiency, and Cytokine−cytokine receptor interaction.

### 3.5 Identification of characteristic genes by machine learning

**Figure 6:**
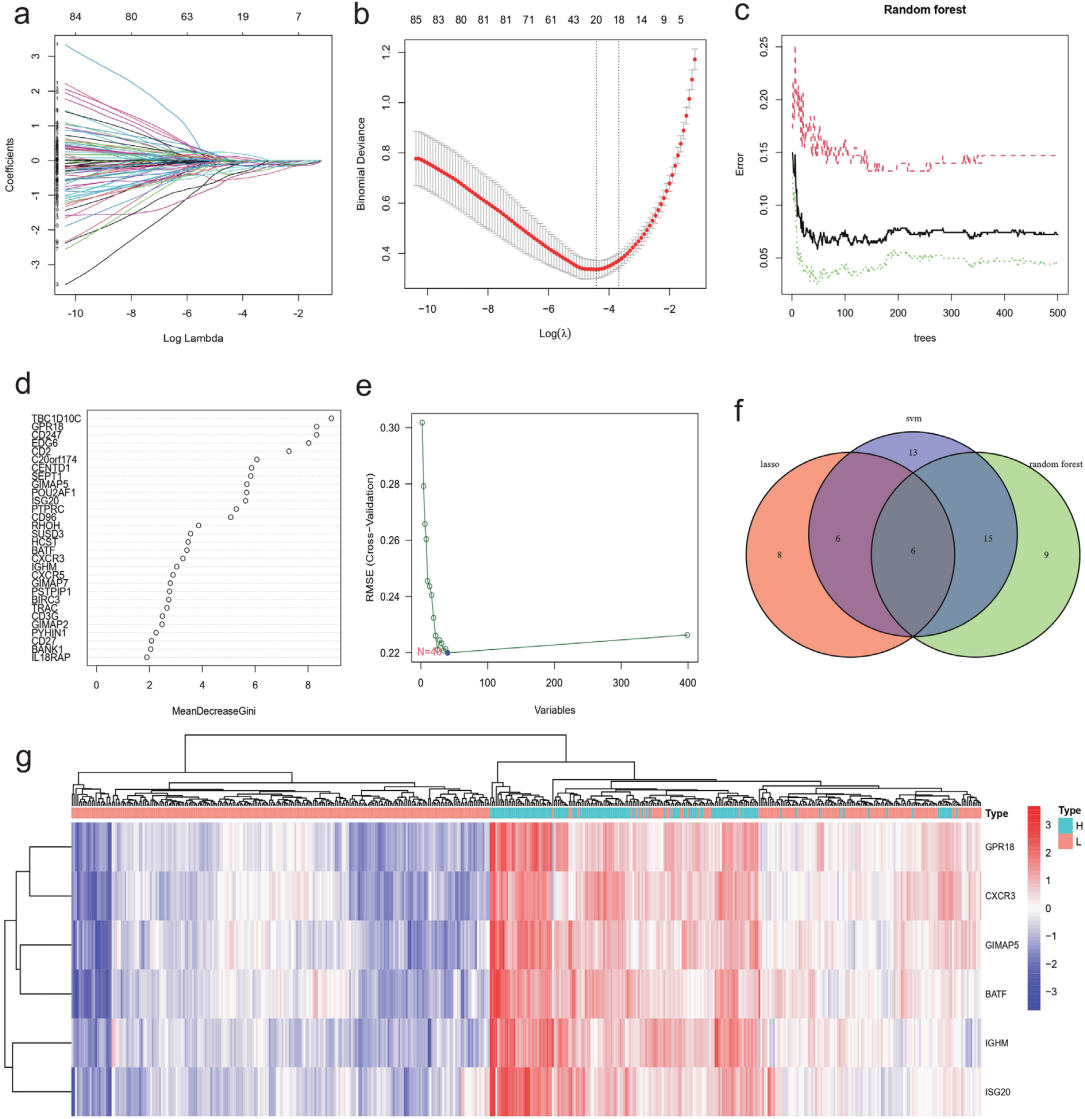
Identification of characteristic gene using machine learning. The Lasso algorithm identified 20 characteristic genes that distinguish the immune levels of NBL: **(A)** regression coefficients plot, **(B)** cross-validation plot. Top 30 characteristic genes ranked by random forest algorithm: **(C)** Out-Of-Bag (OOB) errors against number of trees plot, **(D)** Feature importance plot. **(E)** 40 genes were identified by SVM-RFE algorithm. **(F)** Venn diagram showing the 6 characteristic genes co-identified by LASSO, Random Forest, and SVM-RFE. **(G)** Heatmap of characteristic genes expression.

Machine learning algorithms were employed to identify characteristic genes associated with NBL immunity among the candidate genes screened through differential expression analysis and WGCNA analysis. A total of 20 genes were identified by the LASSO algorithm, which are IGHM, GPR18, CXCR3, etc (**Figures 6A, B**). The top 30 genes ranked by Random Forest were TBC1D10C, GPR18, CD247, etc (**Figures 6C, D**). In the the SVM-RFE algorithm, the error reached its lowest value when the feature count was 40, comprising GPR18, ARHGAP9, C20orf174, etc (**Figure 6E**). The Venn diagram results (**Figure 6F**) showed that a total of six overlapping genes (BATF, CXCR3, GIMAP5, GPR18, ISG20, IGHM) were identified by the three machine learning algorithms and all six genes were up-regulated in the high immunity group (**Figure 6G**).

### 3.6 Diagnostic capability of characteristic genes for NBL immunoclustering

**Figure 7:**
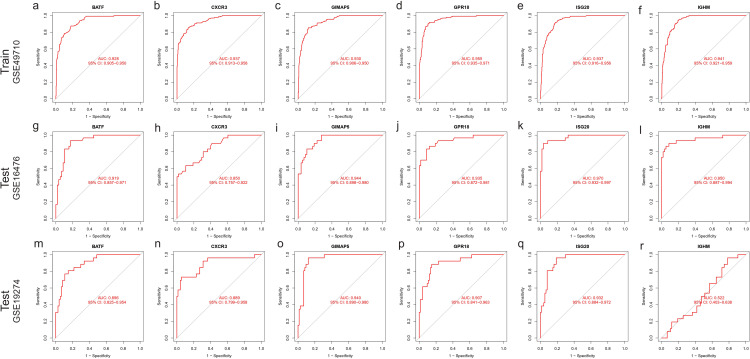
ROC curve of characteristic gene. Train dataset GSE49710: **(A)** BATF, **(B)** CXCR3, **(C)** GIMAP5, **(D)** GPR18, **(E)** ISG20, **(F)** IGHM. Test dataset GSE16476: **(G)** BATF, **(H)** CXCR3, **(I)** GIMAP5, **(J)** GPR18, **(K)** ISG20, **(L)** IGHM; Test dataset GSE16476: **(M)** BATF, **(N)** CXCR3, **(O)** GIMAP5, **(P)** GPR18, **(Q)** ISG20, **(R)** IGHM.

First, we analyzed the diagnostic ability of six characteristic genes for immunoclustering of NBL in the training dataset by plotting ROC curves. The analysis results suggested that all six characteristic genes could serve as diagnostic markers for NBL immunoclustering (BATF: AUC: 0.928, 95%CI: 0.905−0.950; CXCR3: AUC: 0.937, 95%CI: 0.913−0.958; GIMAP5: AUC: 0.930, 95%CI: 0.906−0.950; GPR18: AUC: 0.955, 95%CI: 0.935−0.971; ISG20: AUC: 0.937, 95%CI: 0.916−0.956; IGHM: AUC: 0.941, 95%CI: 0.921−0.959, **Figures 7A–F**). When analyzing the GSE16476 test dataset, all the characteristic genes exhibited the same trend as in the train dataset (BATF: AUC: 0.919, 95%CI: 0.857−0.971; CXCR3: AUC: 0.850, 95%CI: 0.757−0.922; GIMAP5: AUC: 0.944, 95%CI: 0.898−0.980; GPR18: AUC: 0.935, 95%CI: 0.872−0.981; ISG20: AUC: 0.970, 95%CI: 0.932−0.997; IGHM: AUC: 0.950, 95%CI: 0.887−0.994, **Figures 7G–L**). In addition, when analyzing the GSE19274 test dataset, we found that all genes had satisfactory diagnostic capabilities (BATF: AUC: 0.896, 95%CI: 0.825−0.954; CXCR3: AUC: 0.889, 95%CI: 0.799−0.958; GIMAP5: AUC: 0.940, 95%CI: 0.890−0.980; GPR18: AUC: 0.907, 95%CI: 0.841−0.963; ISG20: AUC: 0.932, 95%CI: 0.884−0.972; **Figures 7M–Q**) except for IGHM (AUC: 0.522, 95%CI: 0.403−0.638; **Figure 7R**).

### 3.7 Correlation between characteristic genes and immune cells

**Figure 8:**
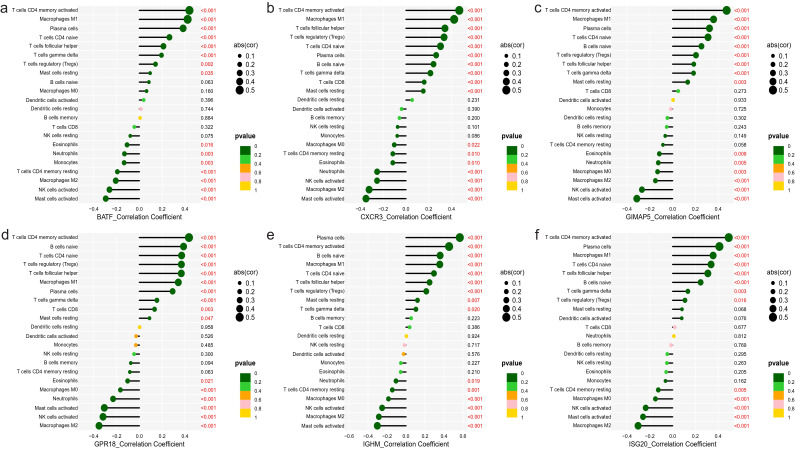
Correlation between characteristic genes and immune cells. **(A)** BATF, **(B)** CXCR3, **(C)** GIMAP5, **(D)** GPR18, **(E)** IGHM, **(F)** ISG20.

We further analyzed the relationship between six characteristic genes and immune cells in NBL. The analysis revealed that BATF was positively correlated with T cells CD4 memory activated, *etc.*, and negatively correlated with Macrophages M2, *etc.*, (**Figure 8A**). CXCR3 had a significant positive association with CD4 memory-activated T cells, *etc.*, and a negative association with Mast cells activated, *etc.*, (**Figure 8B**). GIMAP5 was significantly positively correlated with T cells CD4 memory activated, *etc.*, and negatively correlated with Mast cells activated, *etc.*, (**Figure 8C**). GPR18 was found to be positively correlated with T cells CD4 memory activated, *etc.*, and negatively correlated with Macrophages M2, *etc.*, (**Figure 8D**). IGHM was positively correlated with Plasma cells, *etc.*, and negatively correlated with Mast cells activated, *etc.*, (**Figure 8E**). ISG20 was positively associated with T cells CD4 memory activated, *etc.*, and negatively associated with Macrophages M2, *etc.*, (**Figure 8F**).

### 3.8 Functional enrichment analysis of characterized genes

**Figure 9:**
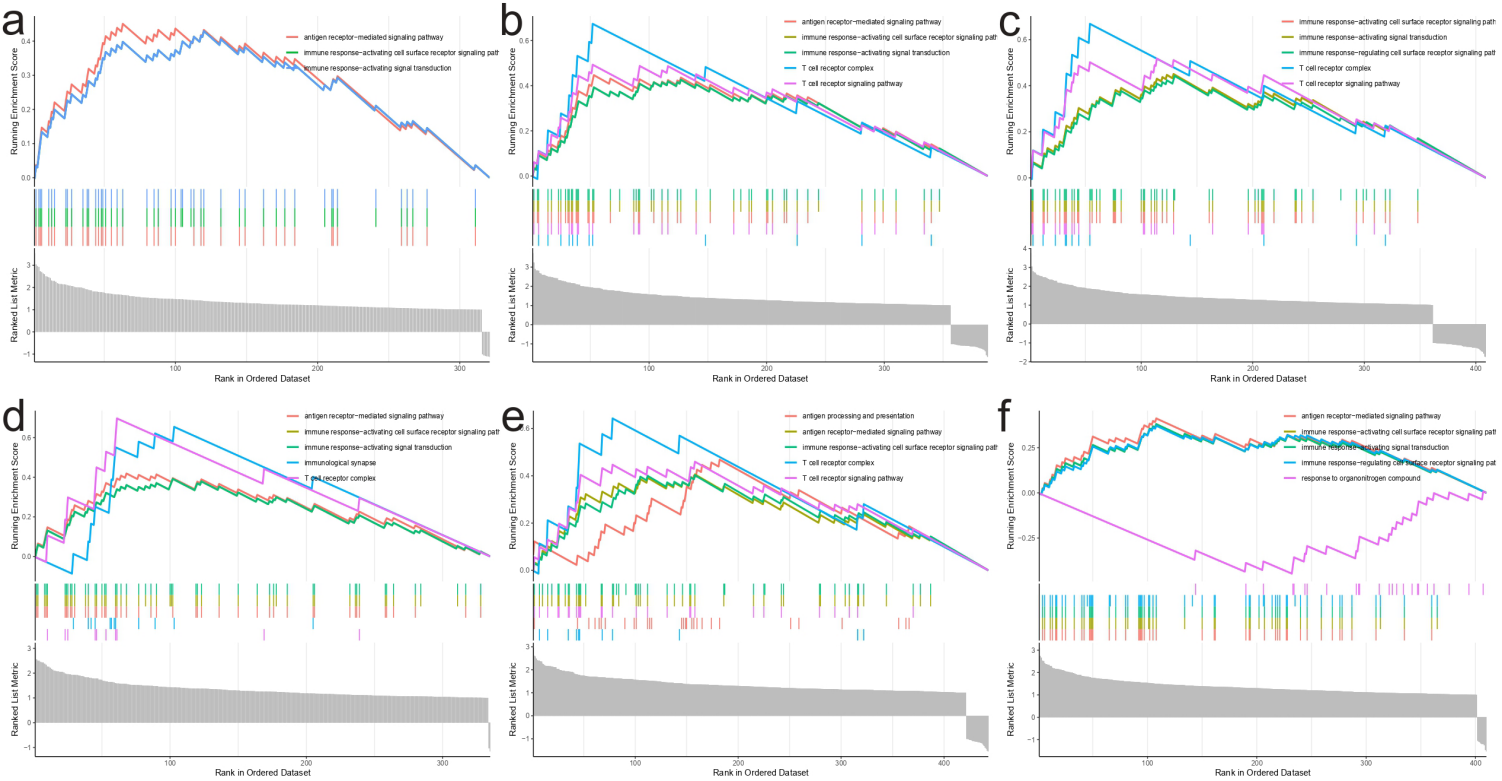
GSEA_GO analysis results. **(A)** IGHM, **(B)** GPR18, **(C)** CXCR3, **(D)** ISG20, **(E)** GIMAP5, **(F)** BATF.

In order to better understand the function of the characterized genes in NBL, we divided the training group into high and low expression groups with the median gene expression value as the threshold, and then performed GSEA_GO analysis on the differentially expressed genes among these two groups. We found that these genes participated in various immune-associated pathways (IGHM: *ie.* antigen receptor−mediated signaling pathway; GPR18: *ie.* antigen receptor−mediated signaling pathway; CXCR3: *ie.*immune response−activating cell surface receptor signaling pathway; ISG20: *ie.* antigen receptor−mediated signaling pathway; GIMAP5: *ie.* antigen processing and presentation; BATF: *ie.* antigen receptor−mediated signaling pathway **Figure 9**).

### 3.9 The effect of characteristic genes on clinical features

We first analyzed the effect of five characteristic genes on the prognosis of NBL patients (There was no expression information for the IGHM gene in GSE85047 dataset). The Kaplan-Meier curve demonstrated that increased expression of the ISG20 gene was negatively associated with the prognosis of NBL patients (OS: P=0.036, PFS: P=0.022). There is no association between the expression of five other characterized genes and the survival of NBL patients (**Supplementary Figure S1**). In addition, we analyzed the correlation between immune-related characteristic genes and clinicopathological features of NBL based on training dataset (GSE49710). The results indicated that IGHM was highly expressed in female patients (**Supplementary Figure S2A**), while CXCR3 and GPR18 were highly expressed in patients younger than 1.5 years (**Supplementary Figure S2B**). We subsequently investigated the relationship between the state of MYCN, an independent prognosticator of NBL, and the expression of characteristic genes. The results showed that all the characteristic genes were downregulated in NBL patients with MYCN amplification (**Supplementary Figure S2C**). BATF, CXCR3, GIMAP5, GPR18, IGHM have lower expression in high-risk neuroblastoma patients (**Supplementary Figure S2D**). Except for IGHM and ISG20, the other characteristic genes were associated with NBL INSS stage, and class label (**Supplementary Figures S2E, F**). We also found that CXCR3 and GPR18 were downregulated in NBL patients with progression (**Supplementary Figure S2G**).

## 4 Discussion

The relationship between NBL and immunity is a complex and intricate one, due to the unique challenges posed by the cold tumor microenvironment of NBL (42). Although considered cold tumors, certain subgroups of NBL exhibit high immune infiltration, possibly due to variations in genetic or epigenetic factors, immune cell composition, and tumor microenvironment (43). These differences underscore the importance of identifying diagnostic biomarkers for effective patient stratification and personalized treatment strategies. Immunotherapy, which leverages the immune system to recognize and destroy cancer cells, has emerged as a promising treatment option for various cancers (44). However, its efficacy in NBL remains suboptimal, necessitating further investigation into the underlying mechanisms and potential therapeutic targets (45).

In this study, we analyzed data from the GEO database and performed immunophenotyping using the ssGSEA method to gain insights into the immune landscape of NBL. We employed machine learning algorithms to screen six genes, BATF, CXCR3, GIMAP5, GPR8, IGHM, and ISG20, that have been extensively investigated in other cancers, including lymphoma, melanoma, leukemia, breast cancer, multiple myeloma, hepatocellular carcinoma, and lung cancer. Despite their diagnostic value and clinical significance in other cancers, the roles of these biomarkers in NBL remain largely unexplored.

Our study delved into the functions and associations of these biomarkers with specific immune cell populations in NBL. We found that these biomarkers were mainly positively correlated with tumor-resistant immune cells (e.g., T cells, macrophages M1, etc.) and negatively correlated with tumor-promoting immune cells (e.g., macrophages M2, etc.) (46). This suggests that these biomarkers may play a crucial role in shaping the immune landscape in NBL, ultimately influencing the balance between antitumor immunity and immune evasion. For instance, BATF has been studied extensively in lymphoma and melanoma and is known to regulate T helper (Th) cell differentiation and immune responses (47, 48). In NBL, BATF might regulate Th cell differentiation, thereby shaping the immune landscape and influencing the immune response against tumor cells. CXCR3, which has been investigated in various cancers, including colorectal cancer, melanoma, and renal cell carcinoma, directs the migration of immune cells to the tumor site and has been associated with prognosis (49, 50). CXCR3 might contribute to recruiting cytotoxic T cells and natural killer cells to the tumor microenvironment, thus enhancing the immune response against NBL cells and affecting tumor growth and metastasis (51). GIMAP5 has been linked to the survival and homeostasis of lymphocytes and may influence the clinical outcome in leukemia (52). GIMAP5 might regulate T cell development and function within the tumor microenvironment, possibly affecting the survival and function of immune cells and the overall immune response against NBL cells (53). GPR8, which has been linked to the regulation of cell growth and proliferation in breast cancer, might regulate signaling pathways crucial for cancer cell survival and metastasis, as well as influence the immune microenvironment by modulating immune cell recruitment and activation in NBL (54, 55). IGHM, associated with B-cell receptor signaling, might modulate the immune response by promoting B cell activation and antibody production in NBL, which could influence tumor progression and response to therapy (56, 57). Lastly, ISG20, which has been studied in hepatocellular carcinoma and lung cancer, may play a role in modulating the type I interferon response and activating immune cells crucial for antitumor immunity in NBL (58, 59).

Current treatment options for NBL, such as chemotherapy, radiation therapy, and immunotherapy, often result in suboptimal prognosis (60). Precision medicine and effective patient stratification are essential for improving therapeutic outcomes. Our study found that treatment effectiveness in NBL patients was highly correlated with the level of immune infiltration, emphasizing the importance of understanding the immune landscape in NBL for better patient management. Moreover, we conducted drug sensitization analyses that could potentially inform personalized treatment strategies for patients. In addition to conventional chemotherapy, immunotherapy has shown promise in other solid tumors. For example, pembrolizumab, a PD-1 inhibitor, has significantly prolonged survival in non-small cell lung cancer (60). This success has been attributed to the targeting of immune checkpoint molecules, such as PD-1, which play a crucial role in regulating immune responses and maintaining self-tolerance (61). Pembrolizumab has become a mature second-line drug in clinical guidelines for non-small cell lung cancer, and numerous meta-analyses and systematic reviews have shown clear evidence of its ability to prolong survival compared to other treatments (62). However, the development of immunotherapy in NBL is less advanced compared to other solid tumors, such as gastric cancer and lung cancer. The identification of novel immunotherapeutic targets and strategies is crucial for improving treatment outcomes in NBL. Our study contributes to the growing body of evidence supporting the potential of immunotherapy in NBL by exploring the immune landscape and identifying potential diagnostic biomarkers. These findings provide a foundation for future research and the development of more effective and targeted treatments for this devastating pediatric cancer.

## 5 Conclusion

In conclusion, this study underscores the importance of understanding the relationship between NBL and immunity, and the potential role of immunotherapy as a treatment option. By analyzing the immune landscape of NBL, we identified six genes - BATF, CXCR3, GIMAP5, GPR8, IGHM, and ISG20-with diagnostic and clinical significance in other cancers, but whose roles in NBL remain largely unexplored. Our research delved into the functions and associations of these biomarkers with specific immune cell populations in NBL, uncovering positive correlations with immune-positive cells and negative correlations with immune-negative cells. Furthermore, our findings suggest that the level of immune infiltration is crucial for treatment effectiveness in NBL patients, and drug sensitization analyses may aid in developing personalized treatment strategies.

## Data Availability

The original contributions presented in the study are included in the article/**Supplementary Material**. Further inquiries can be directed to the corresponding authors.

## Supplementary material

Supplementary Figure 1 Effects of characteristic gene expression on survival in NBL patients using Kaplan-Meier curve analysis. OS: **(A)** BATF, **(B)** CXCR3, **(C)** GIMAP5, **(D)** GPR18, **(E)** ISG20. PFS: **(F)** BATF, **(G)** CXCR3, **(H)** GIMAP5, **(I)** GPR18, **(J)** ISG2.

Supplementary Figure 2 Relationship between the expression of characteristic genes and clinicopathologic features of NBL. **(A)** Sex, **(B)** Age, **(C)** MYCN status, **(D)** High risk, **(E)** INSS stage, **(F)** Class label, **(G)** Progression. (Note: High risk: Clinically considered as high-risk neuroblastoma. INSS stage: disease stage according to INSS. Class label: Maximally divergent disease courses - Favorable: patient survived without chemotharapy for at least 1000 days post diagnosis; Unfavorable: patient died despite intensive chemotherapy. Progression: Occurrence of a tumor progression event).
