# Supplementary material for: Identification of novel markers for neuroblastoma immunoclustering using machine learning
Source: Front Immunol. 2024 Nov 4;15:1446273. doi: 10.3389/fimmu.2024.1446273 (PMC11570813; doi:10.3389/fimmu.2024.1446273)
Supplement: Supplementary file 1 [file DataSheet1.zip › Supplementary Table S1.DOCX]

Supplementary Material

Supplementary Table

Table S1. Clinicopathological Characteristics of the Immunity_L and Immunity_H Groups

|  | Immunity_L | Immunity_H | p-value |
| --- | --- | --- | --- |
| Age (Days, M±SD) | 731.7±1083.4 | 830.1±876.4 | 0.002 |
| Sex, n (%) |  |  | 0.839 |
| Male | 210 (58.0) | 77 (56.6) |  |
| Female | 152 (41.9) | 59 (43.4) |  |
| MYCN status, n (%)^$^ |  |  | 0.002 |
| Amplified | 79 (21.8) | 13 (9.6) |  |
| No amplification | 280 (77.3) | 121 (89.0) |  |
| No information | 3 (0.8) | 2 (1.5) |  |
| High risk, n (%)^*^ |  |  | 0.036 |
| Yes | 138 (38.0) | 38 (28.0) |  |
| No | 224 (62.0) | 98 (72.0) |  |
| INSS stage, n (%)^£^ |  |  | 0.028 |
| 1 | 78 (21.5) | 43 (31.6) |  |
| 2 | 54 (14.9) | 24 (17.6) |  |
| 3 | 49 (13.5) | 14 (10.3) |  |
| 4 | 135 (37.3) | 48 (35.3) |  |
| 4S | 46 (12.7) | 7 (5.1) |  |
| Class label, n (%)^#^ |  |  | 0.163 |
| Favorable | 122 (33.7) | 59 (43.4) |  |
| Unfavorable | 69 (19.1) | 22 (16.2) |  |
| N/A | 171 (47.2) | 55 (40.4) |  |
| Progression, n (%)^&^ |  |  | 0.348 |
| Yes | 138 (38.1) | 45 (33.0) |  |
| No | 224 (61.9) | 91 (66.9) |  |

^$^Samples without information(No information) were not statistically analyzed.

^*^High risk: Clinically considered as high-risk neuroblastoma.

^£^INSS stage: disease stage according to International Neuroblastoma Staging System (INSS).

^#^Class label: Maximally divergent disease courses - Favorable: patient survived without chemotharapy for at least 1000 days post diagnosis; Unfavorable: patient died despite intensive chemotherapy; not applicable (N/A). Samples without information(N/A) were not statistically analyzed.

^&^Progression: Occurrence of a tumor progression event.
