# Supplementary figures and images for: Identification of novel markers for neuroblastoma immunoclustering using machine learning
Source: Front Immunol. 2024 Nov 4;15:1446273. doi: 10.3389/fimmu.2024.1446273 (PMC11570813; doi:10.3389/fimmu.2024.1446273)

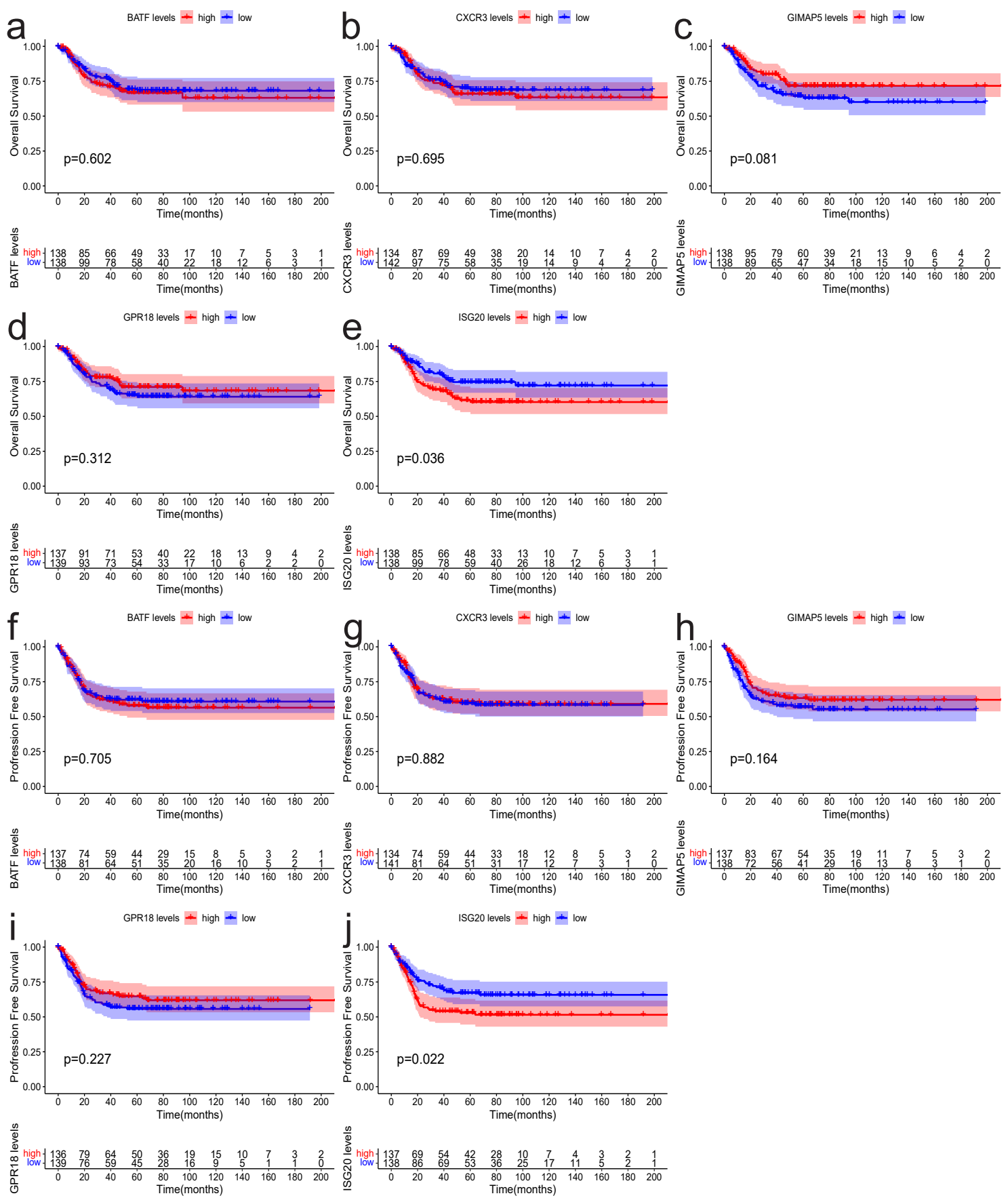

Supplement: Supplementary file 1 [file DataSheet1.zip › Supplementary Figure S1.PDF]

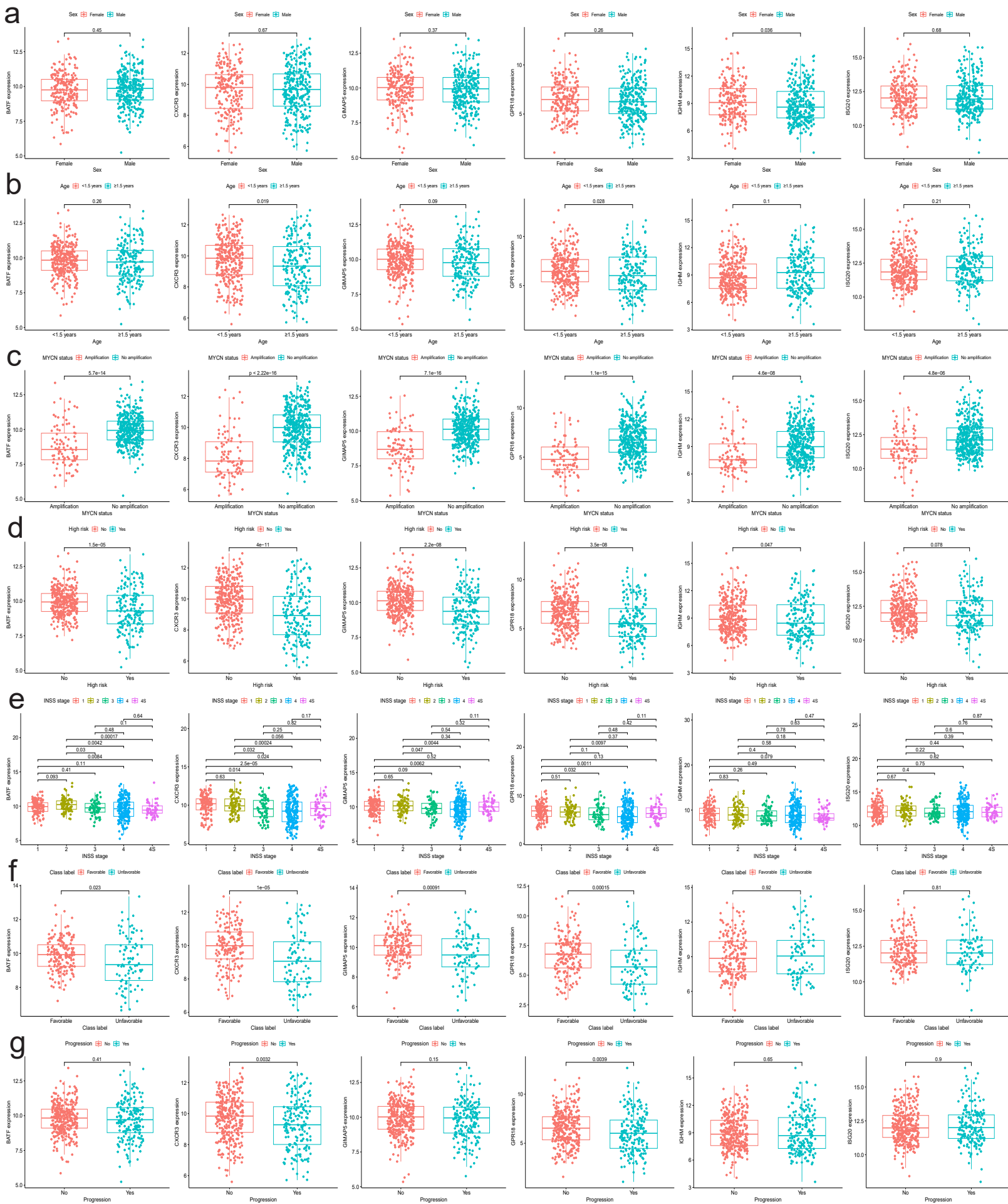

Supplement: Supplementary file 1 [file DataSheet1.zip › Supplementary Figure S2.PDF]
